# Energy Analysis of a Complementary Heating System Combining Solar Energy and Coal for a Rural Residential Building in Northwest China

**DOI:** 10.1155/2018/2158205

**Published:** 2018-02-14

**Authors:** Xiaofei Zhen, Jinping Li, Yassir Idris Abdalla Osman, Rong Feng, Xuemin Zhang, Jian Kang

**Affiliations:** ^1^Western China Energy & Environment Research Center, Lanzhou University of Technology, Lanzhou 730050, China; ^2^Key Laboratory of Complementary Energy System of Biomass and Solar Energy, Lanzhou, Gansu Province 730050, China; ^3^Collaborative Innovation Center of Key Technology for Northwest Low Carbon Urbanization, Lanzhou 730050, China; ^4^Shaanxi Key Laboratory of Industrial Automation, Shaanxi University of Technology, Hanzhong 723000, China

## Abstract

In order to utilize solar energy to meet the heating demands of a rural residential building during the winter in the northwestern region of China, a hybrid heating system combining solar energy and coal was built. Multiple experiments to monitor its performance were conducted during the winter in 2014 and 2015. In this paper, we analyze the efficiency of the energy utilization of the system and describe a prototype model to determine the thermal efficiency of the coal stove in use. Multiple linear regression was adopted to present the dual function of multiple factors on the daily heat-collecting capacity of the solar water heater; the heat-loss coefficient of the storage tank was detected as well. The prototype model shows that the average thermal efficiency of the stove is 38%, which means that the energy input for the building is divided between the coal and solar energy, 39.5% and 60.5% energy, respectively. Additionally, the allocation of the radiation of solar energy projecting into the collecting area of the solar water heater was obtained which showed 49% loss with optics and 23% with the dissipation of heat, with only 28% being utilized effectively.

## 1. Introduction

In rural areas of northern China, the energy consumption for heating buildings has reached 1 × 10^9^ tce, which occupies a large portion of the 56% of the total energy consumption, of which coal comprises 74% [1]. It is expected that, with the development of the economy in rural areas and the improvement of the living standards of farmers, the energy consumption will increase tremendously in the future, which is detrimental to the sustainable development of a society as a whole. The utilization of the local renewable energy resources that are replacing fossil-fuel energy to meet the heating demands of buildings is becoming an effective method of solving this problem. Therefore, solar energy has long been one of the most abundant alternative resources arousing wide public interest.

### 1.1. Literature Review

It is widely known that there are two major types of solar heating technology, namely, passive and active. Passive solar energy technology is the earliest pattern for heating buildings by the utilization of solar energy, which uses building orientation, glass windows, and other building materials for collecting, storing, and utilizing solar energy to achieve indoor thermal comfort [2, 3]. In 1977, the first passive solar house in China was constructed in Minqin County, Gansu Province. A large number of studies have been performed on the performance improvements of this technology. Barea et al. [4] investigated and calculated the thermal performance of a specific orientation of multiazimuthal windows. They concluded that multiazimuthal windows with an angle of 45° in the side panels reach a multiazimuthal/flat solar gain factor (M/FSGF) daily average value of 1.20, which means 20% more solar gain than the daily average solar gain of a flat window. In addition, a window with an angle of 90° in the side panels is the best for temperate climates, with daily average solar gains 27% greater than those of flat windows. Tiwari and Sahota [5] presented a review on the energy and economic efficiencies of passive and active solar distillation systems, highlighted by experimental and theoretical detailed work performed in the recent past on passive and active solar technology. Jie et al. [6] studied the indoor temperature of a fenestrated room with PV-Trombe walls. Rempel et al. [7] found that over half of all the energy input into each sunspace originated as diffuse solar radiation. Nguyen and Reiter [8] examined the potential of improving thermal comfort under the climatic conditions of Vietnam resulting from a passive strategy by using a newly developed climate analysis tool. Wang et al. [9] found that a passive solar house equipped with a water thermal storage wall reduced yearly energy consumption by 8.6%. In addition, many recent studies have focused on the phase-change materials integrated in passive solar houses [10–12]. Mazarrón et al. [13] studied the design, installation, and evaluation of a solar water heating system with an evacuated tube collector and active circulation. The results showed that the energy collected can be increased sharply by improving the collector characteristic and reducing the pipes losses. The kang is a primary heating method in rural areas of northwest China. According to the characteristics of a traditional fire-based kang, Jiang et al. [14] presented a theoretical study of a solar-based kang system, the results of which are in accordance with experiment. Bellos et al. [15] suggested an innovative Trombe wall as a passive heating system for a building.

The solar-assisted heat pump is another important active solar heating technology that has attracted much attention. Han et al. [16] proposed a multisource hybrid heat pump system (MSHHPS) with seasonal thermal storage. The composition, operation modes, mathematical model, and transition conditions of the different operating modes of the system were determined. Ma et al. [17] presented an evaluation of the performance of a solar-groundwater heat pump (SGHP) unit associated with radiant floor heating. The results show that the new system has an energy-saving rate of 30.55% compared to a conventional central heating system (CCHS). The floor heating systems can have an energy-saving rate of 18.96% compared to a traditional radiator. Deng and Yu [18] investigated a combined solar/air, dual-source, heat pump water-heater system for domestic water heating applications. The simulation results show the modified direct-expansion solar-assisted heat pump water heaters (M-DX-SHPWHs) system exhibits better performance than that of a conventional direct-expansion solar-assisted heat pump water heaters (DX-SHPWH) system. At a low solar radiation rate of 100 W/m^2^, the M-DX-SHPWH heating time decreases by 19.8% compared to the DX-SHPWH when the water temperature reaches 55°C. Meanwhile, the COP, on average, increases by 14.1%. Ni et al. [19] projected a phase-change-material- (PCM-) based, solar-assisted air-source heat pump (PCM-SAHP) system consisting of an air-source heat pump (ASHP), a PCM unit, and a solar thermal collector. The experimental findings indicate that in the cooling mode the ambient temperature still has a significant effect on the operating performance of the system. In contrast, it was found that the effect of the chilling water flow rate (throughout the PCM unit) on the system performance is relatively subtle. Fraga et al. [20] addressed the analysis of the potential of a combined solar thermal and heat pump (HP) system on new and existing multifamily buildings in which a numerical simulation as a complement to a monitored study case was proposed. Chen et al. [21] experimentally and theoretically investigated a solar combination system consisting of a solar collector and a CO_2_ heat pump. The simulated results show that the optimized system can save 14.2% of electricity and improve the solar fraction by 8%, with the solar fraction of the optimized system capable of reaching 71.1%. Kong et al. [22] described a direct-expansion solar-assisted heat pump (DX-SAHP) system that uses R410A as a refrigerant, which can supply domestic hot water over the entire year, and provided a numerical model to estimate the thermal performance of the system.

There have also been many studies related to innovative solar heating systems. Chen et al. [23, 24] designed a nearly net-zero annual energy consumption house in Eastman, Quebec, Canada, utilizing building-integrated thermal mass both in passive and in active forms, and the annual space heating energy consumption of the house was approximately 5% of the national average. A new type of radiant end system with even lower supplying temperature for a solar or solar-assisted space heating system was proposed in Ren et al. [25], and the simulation results showed that the new end system had good prospects for the effective use of local renewable resources. Esen and Yuksel [26] conducted an experimental study to investigate greenhouse heating by biogas, solar, and ground energy in Elazig. Yu et al. [27] proposed a hybrid solar air heating system for building space heating, which included passive and active dual-function solar collectors. Detailed measurements of the auxiliary energy consumption for space heating and domestic hot water preparation in two detached passive houses heated by a solar water heating system and an air-to-water heat pump were reported in [28].

Economic analysis has been performed for solar heating systems as well. Ziemele et al. [29] examined the transition from a conventional district heating (DH) system to a fourth-generation DH (4GDH) system using system dynamics modeling and economic feasibility analysis. The results show how the price of a fossil-fuel influences the share of heat energy production and the balance point between investment at the source and on the heat consumer side. Milani et al. [30] addressed the proposition of the hybridization concept and the simulation of benchmark power plants for a suitable Brazilian site (high direct normal irradiation and low-cost biomass availability). Sonthikun et al. [31] designed and constructed a solar-biomass hybrid for natural rubber sheet drying. The solar-biomass hybrid dryer was tested for drying of 100 natural rubber sheets, and the moisture content of the rubber sheets was reduced from 34.26% to 0.34% (dB) in only 48 h. Mehdaoui et al. [32] presented a parametric study with the help of the TRNSYS program to optimize solar heating prototype design parameters, and the program was validated by experiment.

Recently, numerous rural buildings are being built in northwest China; however, the majority of them lack solar heating technology since they are located in areas where coal is still the primary heating resource. Although many renewable systems have appeared, few studies have focused on rural residential buildings in cold regions of northwest China. Those buildings have unique characteristics: scattered distribution and weak energy-conservation performance dominate; however, sufficient space exists for installation of solar energy utilization devices, and it is easy to utilize local biomass energy resources.

## 2. Description of the Rural Household Building and the Complementary Heating System

A complementary heating system combining solar energy and coal for a rural household building has been established in a new socialist countryside called Zhangma, Minqin County, Gansu Province (latitude 38°34′N, longitude 103°3′E). Neither the solar energy utilization device nor the coal stove in the system is highly sophisticated, but they are the easiest to operate and most economical model in practice for the heating of rural household buildings. The experiment on the performance of the system was carried out from December 10, 2014, to March 30, 2015. The aim of the present work was to analyze the efficiency of the energy utilization of the system and to reveal the combined effects of multiple factors on solar energy utilization.

### 2.1. Rural Household Energy-Saving Residential Building

**Figure 1:**
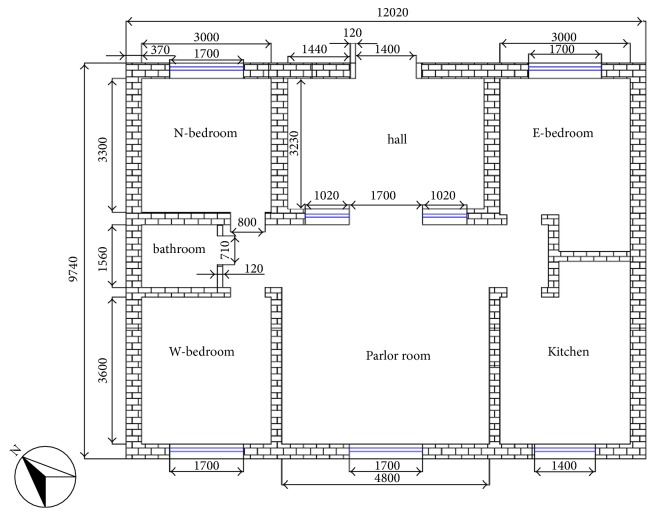
Floor plan of the building (mm).

Figure 1 shows the floor plan of the rural household building under study; the building area is approximately 117 m^2^. The heights of the external walls, the double-glazed windows, and the outer door measurements are 3.3, 1.4, and 3.0 m, respectively. The external wall is constructed of perforated bricks with a thickness of 0.37 m, and the roof is constructed of reinforced concrete with a thickness of 0.15 m. Later, approximately 0.07 m of slag was laid on the roof, as an insulating material. In addition, to decrease the heating loading of the building in winter, extruded polystyrene board (XPS) insulating panels with a thickness of 0.06 m were attached to the outside surface of the external wall, except for the left-side external wall, which is shared with a neighboring structure.

### 2.2. Complementary Heating System

**Figure 2:**
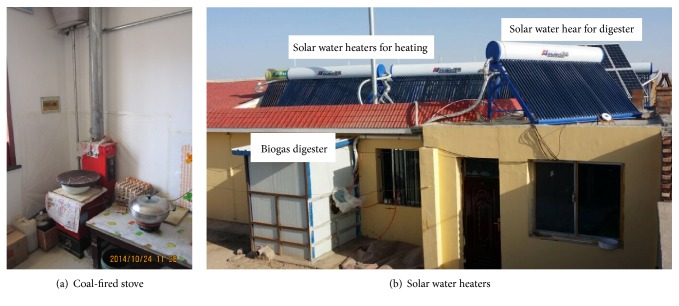
The complementary heating system.

The building's previous heating system was a natural circulation heating system that consisted of a coal stove installed in the kitchen and radiators fixed in other rooms, but not in the hall. Six solar water heaters were added in the new complementary heating system; the existing coal stove and solar water heaters are shown in Figure 2. Each solar water heater has a 3.85 m^2^ collector area (40 all-glass vacuum tubes measuring 0.058 m in diameter and 1.8 m in length) and a 400 L storage tank. The inclination angles of the solar water heaters are all 45°, which is suitable for the optimal inclination angle in local areas. However, the orientation, which is approximately south by west 40°, is not ideal due to the orientation and layout of the building. There are three lines of solar water heaters on the roof of the building, each line consisting of two solar water heaters. After the six solar water heaters were connected in series, they were connected with the coal stove in parallel. A circulation pump was installed in the branch pipe of the series-wound solar water heaters. If the temperature of the water in the storage tank of the solar water heaters is suitable, the circulation pump will deliver the hot water to the radiators to heat the house; the circulation pump will shut down when the temperature of the water is too low to heat the house. The heating system operates like the previous model, with gravity circulation forced by the coal stove.

## 3. Experimental Methods

To analyze the efficiency of the energy utilization of the system, both the input and output energy must be measured or calculated (Using the prototype model, the average thermal efficiency of the furnace is obtained. Based on the measured coal quality, the amount of heat absorbed by the solar energy and released into the room. The energy input to the building is calculated based on the inputs coal and the solar energy.). The intensity of the surface facing south, with an inclined angle of 45° and which receives the maximum solar energy in winter, was measured by a pyranometer. The weight of coal consumed by the stove was also weighed by a platform of the solar radiation on balance every day, and the caloric value of the coal was calculated in the laboratory at Lanzhou University of Technology. The inlet/outlet temperature and the circulation flow rate of the hot water were measured to calculate the energy outputs supplied by the solar water heaters. Moreover, the ambient temperature, the temperature in the five indoor rooms (except the hall), and the temperature of the hot water in one of the storage tanks in the middle line were measured. A solar water heater, used to heat the digester, was employed as a reference that faced south and was not shaded in order to study the influence of the orientation and shade effects on thermal performance. The inlet/outlet temperature, circulation flow rate, and water temperature in the storage tank of the reference solar water heater were also measured. All the measurements were recorded automatically by a data acquisition system (Agilent 349702, Agilent Technologies, USA) every 10 s. The detailed parameters of the measuring instruments used in this study are shown in Table 1.

The energy output supplied by the coal stove was difficult to measure directly, so a prototype model for calculating the thermal performance of the coal stove was set up; it will be described in detail in Section 4.1.

## 4. Results and Discussion

### 4.1. Prototype Model for Calculating the Thermal Efficiency of the Coal Stove

There are several studies that refer to the efficiency of traditional natural circulation coal stoves. Jiang [1] obtained a value of 32% in a field experiment. The results of Yang et al. [33] showed an efficiency of 30–40%. In general, there are two main methods of calculating the thermal efficiency of a coal-fired boiler and stove, namely, the direct balance and indirect balance methods. However, both were unsuitable for this study because the stove was installed in the kitchen. The heat released from the stove's surface cannot be measured directly even though it contributed to space heating. Therefore, a prototype model for calculating the thermal efficiency of the coal-fired stove in this system was proposed.

Chinese National Standard JGJ 132-2001, entitled “Standard for Energy Efficiency Inspection of Heating Residential Buildings” [34], provided a method of measuring the heat loss of a building, which is expressed by

(1) $$\begin{array}{c} q_{hm}=\frac{Q_{hm}}{A_{0}}\cdot \frac{t_{i}-t_{e}}{t_{ia}-t_{ea}}\cdot \frac{278}{H_{r}}+\left(\;\frac{t_{i}-t_{e}}{t_{ia}-t_{ea}}-1\;\right)\cdot q_{IH}, \end{array}$$

where *q*_*hm*_ is the heat loss of a building, in W/m^2^; *Q*_*hm*_ is the total heat entering the building during the experiment, in MJ; *A*_0_ is the heating area of the building, 103 m^2^ (except for the hall); *q*_IH_ is the internal heat liberation rate of the building, 3.8 W/m^2^ [35]; *t*_*i*_ is the designed indoor temperature, 14°C, which is selected according to [36]; *t*_*e*_ is the designed outdoor temperature, −2.6°C [35]; *t*_*ia*_ is the average indoor temperature during the experiment, in °C; *t*_*ea*_ is the average outdoor temperature during the experiment, in °C; and *H*_*r*_ is the experiment duration, in h (the duration of the experiment, 278, is the conversion factor).

From (1), the calculation for a given building in given region requires all the parameters, which can be calculated by experiment in this study under the condition that the solar water heaters work alone, as follows:

(2) $$\begin{array}{c} Q_{hm,s}=\sum cm\left(\;T_{in}-T_{out}\;\right)t, \end{array}$$

where *c* is the specific heat capacity of water, 4200 J/(kg·°C), *m* is the circulation flow rate for building heating, in kg/s; *T*_in_ is the inlet temperature, in °C; *T*_out_ is the outlet temperature, in °C; and *t* is the time interval of each scan by the Agilent 349702 (the scanning interval is 10 s).

When the coal-fired stove is used for heating only, *Q*_*hm*_ marked by *Q*_*hm*,*c*_ can then be calculated using (3), which is transformed from (1). The thermal performance of the coal-fired stove can therefore be obtained:

(3) Qhm,c=qhm−ti−tetia−tea−1·qIH·A0·tia−teati−te·Hr278.

In practice, there are three heating models, namely, (1) using the coal-fired stove as the unique heating source on cloudy days; (2) using the solar water heaters and coal stove together for space heating when it is sunny, but the solar radiation is not sufficient; and (3) using the solar water heaters to supply the total heating energy when the solar energy is sufficient. For model 2, the coal-fired stove is used in the morning and during the time after the solar heating finishes at night. In the daytime, neither the coal stove nor solar water heaters work when the residents leave their home. The solar water heaters begin to provide heat at night according to the practical situation. In order to reduce the impact of the indoor temperature when the sunlight enters the building from the window, five durations are selected from model 2 as shown in Table 2. During the five durations, when the circulating pump starts to work, no sunlight enters the building. Similarly, all seven durations in model 1 are adopted in (3). The detailed durations and results are shown in Table 2. It can be seen in Table 2 that the *q*_*hm*_ calculations are within the range 32.3–44.0 W/m^2^; the corresponding *Q*_*hm*_ range for 1 kg of coal is 5.5-MJ/kg. Some factors may lead to errors. First, Chinese National Standard JGJ 132-2001 [34] requires that the duration for an experiment be no less than 168 h; however, the duration in this study cannot satisfy the requirements due to conditionality. Second, it is very difficult to measure the indoor temperature exactly [34]. In addition, the internal heat gain and the ventilation rate were different from the design values. Other possible issues include the passive energy gain and the operating conditions of the coal stove. The stove cannot work at an optimum performance level when the room is empty. Therefore, the average value of each result is selected to be the final result, namely, 38.1 W/m^2^ for *q*_*hm*_ and 6.4 MJ/kg for *Q*_*hm*_. Then, the caloric value of the coal is 16.7 MJ/kg, and the average thermal efficiency is 38%, which are close to the above-mentioned results. During the experiment period, the accumulated heat that entered the building was 11619 MJ from solar energy and 7571 MJ from coal, which means that the energy portions are 60.5% and 39.5%, respectively.

### 4.2. Heat-Collection Capacity of the Solar Water Heater

In fact, the heat-collection capacity and the efficiency of the solar water heater are influenced by a series of factors, such as solar radiation, ambient temperature, collecting temperature, and orientation. A relational expression for the determination of the daily heat-collection capacity is provided in Chinese National Standard GB/T 18708-2002, which is

(4) $$\begin{array}{c} Q_{s}=a_{1}H+a_{2}\left(\;T_{b}-T_{a}\;\right)+a_{3}, \end{array}$$

where *Q*_*s*_ is the capacity of the daily heat-collection capacity of the storage tank, in MJ; *H* represents the daily solar radiation on the collecting area of solar water heater, in MJ/m^2^; *T*_*b*_ relates to the initial water temperature in the storage tank, in °C; *T*_*a*_ is the average ambient temperature, in °C; and *a*_1_, *a*_2_, and *a*_3_ are the coefficients that must be determined by experiment.

The duration for calculating the heat-collection capacity during the daytime is valid when the solar radiation intensity is higher than 120 W/m^2^; moreover, the circulation pumps may sometimes work in the duration, given that *T*_*b*_ in this study refers to the average water temperature during the daytime.

As mentioned above, a solar water heater at its best working conditions is employed as a reference, and its thermal behavior will be realized using the same method. For convenience, a single solar water heater used for heating the building was designated SI, and the output energy of each solar water heater is considered to be equal; the reference one was designated SII.

SI and SII are calculated by the following:

(5) QSI=∑t1t2cmTin−Toutt6+cMTt1−Tt2,QSII=∑t1t2cm′Tin−Toutt+cMTt1−Tt2,

where *t*_1_ is the time when the solar radiation intensity is greater than 120 W/m^2^; *t*_2_ is the time when the solar radiation intensity is less than 120 W/m^2^; *T*_*t*_1__ is the water temperature in the storage tank at *t*_1_; *T*_*t*_2__ is the water temperature in the storage tank at *t*_2_; *m*′ is the circulation flow rate for the heating digester, in kg/s; and *M* is the weight of the water in a storage tank, 400 kg.

**Figure 3:**
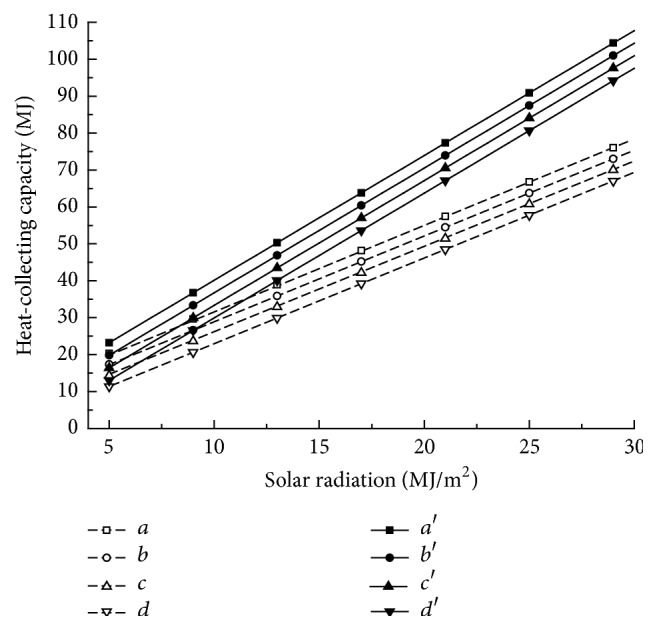
The relationship of heat-collecting capacity between solar radiation and temperature difference. *a*: *t*_*b*_ − *t*_*ad*_ = −10°C; *b*: *t*_*b*_ − *t*_*ad*_ = 0°C; *c* = *t*_*b*_ − *t*_*ad*_ = 10°C; *d* = *t*_*b*_ − *t*_*ad*_ = 20°C; *a*, *b*, *c*, and *d* were for SI; *a*′, *b*′, *c*′, and *d*′ were for SII.

All the related data were dealt with using multiple linear regression models in Excel. The analytical results are calculated using (6) and (7) below.  Figure 3 shows the relationship of heat-collection capacity to the daily solar radiation and the differences in the temperature of the water in and around the storage tank. From these equations and Figure 3, it is obvious that not only is the increasing rate of the heat-collection capacity of SI with daily solar radiation less than that of SII, but its decreasing rate with increasing temperature difference is less as well. Moreover, *R*^2^ of SI is less than that of SII, mainly because of the inconsistency of the water temperature in the six storage tanks caused by the shade between them:

(6) QSI=2.32H−0.30tb−ta+5.75,R2=0.633,

(7) QSII=3.38H−0.34tb−ta+2.94,R2=0.896.

### 4.3. Heat-Loss Coefficient of the Storage Tank

When solar energy is transformed into thermal energy, a part of it is transported into the building for space heating, but another part is lost to the ambient because the water temperature is always higher than the ambient temperature, so it is important to know the heat-loss coefficient of the storage tank. The Chinese National Standard GB/T 18708-2002 also provides an experimental method for determining the heat-loss coefficient of a storage tank, as follows:

(8) $$\begin{array}{c} U_{s}=\frac{cM}{\Delta t}\ln\left(\;\frac{T_{i}-T_{a,av}}{T_{f}-T_{a,av}}\;\right), \end{array}$$

where *U*_*s*_ is the heat-loss coefficient of the storage tank, in W/K; Δ*t* is the time interval of experiment, 28800 s; *T*_*i*_ is the initial water temperature, in °C; *T*_*f*_ is the final water temperature, in °C; and *T*_*a*,av_ is the average ambient temperature, in °C.

The following experimental conditions are required by Chinese National Stand GB/T 18708-2002: Static Conditions; that is, the uniform temperature field being kept above 50°C; natural cooling for 8 h and nine uniform temperature points to calculate; and an average wind speed of less than 4 m/s. Six durations in all were in accordance with these conditions, and the results obtained are shown in Table 3.

The calculated values ranged from 3.59 to 4.70 W/K, and the error might have resulted from some unmeasured parameters, such as the wind speed. The average value of 4.10 W/K is therefore taken as the heat-loss coefficient of the storage tank. Otherwise, another Chinese National Standard, GB/T 19141-2011 [37], establishes the rule that the average heat-loss factor (*U*_*sl*_) of domestic solar water heating systems must be under 16 W/(m^3^·K). When transforming the obtained *U*_*s*_ to *U*_*sl*_ using (7), the value is 10.25 W/(m^3^·K), which meets the requirement of the Chinese National Standard GB/T 19141-2011:

(9) $$\begin{array}{c} U_{sl}=\frac{U_{s}}{V}, \end{array}$$

where *V* is the volume of the storage tank, 0.4 m^3^.

### 4.4. Allocation of Solar Energy

The energy-conservation equation for a single solar water heater can be expressed as follows:

(10) $$\begin{array}{c} Q_{in}=Q_{u}+Q_{h,l}+Q_{h,o}+Q_{st}, \end{array}$$

where *Q*_in_ is the solar energy projected onto the collecting area of a solar water heater, *Q*_*u*_ the heat energy utilized effectively, *Q*_*h*,*l*_ the heat loss, *Q*_*h*,*o*_ the optical loss, and *Q*_st_ the energy stored in the water, which can be neglected for the duration of the long-time experiment period. The detailed method of calculating *Q*_in_, *Q*_*u*_, and *Q*_*h*,*l*_ is as follows:

(11) Qin=∑∑t1t2IAt,

(12) Qu=∑cmΔTin−Tout,

(13) Qh,l=∑UlT−Tamt,

where *I* is the solar radiation intensity, in W/m^2^, and *T* is the transient water temperature in the storage tank, in °C. Then, *Q*_*h*,*o*_ can be determined as follows:

(14) $$\begin{array}{c} Q_{h,o}=Q_{in}-Q_{u}-Q_{h,l}. \end{array}$$

**Figure 4:**
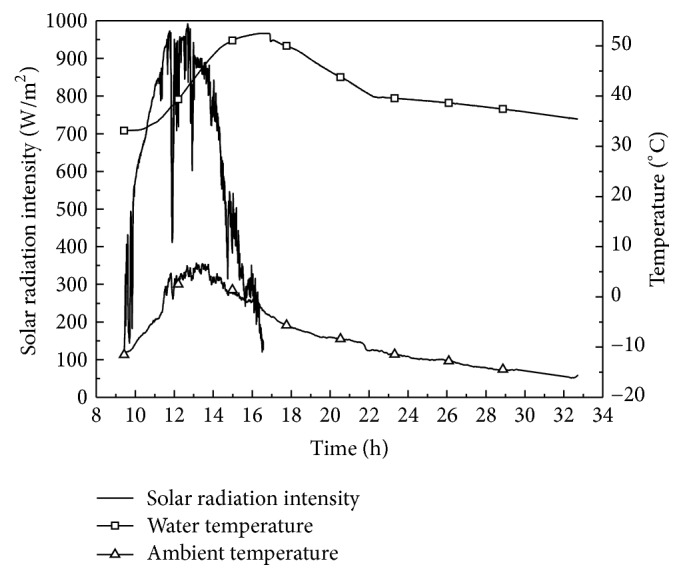
The variations of water temperature, ambient temperature, and solar radiation intensity of a typical duration.

Using the above equations, the allocation of the solar energy projected onto the collector area of a solar water heater for heating the building during the experiment period was obtained.  Figure 4 shows the variation of the temperature of the water in the storage tank, the ambient temperature, and the intensity of the solar radiation for a typical duration, which started at *t*_1_ on December 14, 2015 and finished at *t*_1_ on December 15, 2015. The duration of the sunshine on December 14 was 7.2 h, and the total solar energy radiation projected onto the collector area of a solar water heater was 63.1 MJ. The water temperature in the storage tank was 33.1°C at the beginning and reached its peak point of 52.5°C at 16:45, when the circulation pump started to work. Later, the temperature decreased sharply because of the heat energy not only being transferred into the building, but also being released into the environment. At 22:15 the rate of change decreased gradually, when the circulation pump was shut down and the water temperature was 40°C. Based on (4), (10), and (11), the results were 32.6, 12.5, and 17.9 MJ, respectively, for the aforementioned duration.

The total radiation of solar energy projected onto a solar water heater was 7046 MJ, of which 49% is lost to optics and 23% to heat dissipation, with only 28% being utilized efficiently. When compared to the reference solar water heater, the portion of optical loss was only 33% in the same period, which shows that orientation and shade have the largest influence on the heat-collection capacity of a solar water heater.

In our view, there are two main factors that cause the high heat loss. The first is the location of the storage tank. Although the heat-loss coefficient is lower than that required by the Chinese National Standard, the storage tank always loses heat to the environment because the water temperature is higher than the ambient temperature. The second factor causing the high heat loss is the mismatch between the working temperature of the radiator and the collecting temperature of the solar water heater. Therefore, when the water temperature is too low to heat the building, the circulation pump will be shut down, and the heat energy cannot then be utilized, but only released to the environment.

## 5. Conclusions and Future Study Recommendations

The energy utilization efficiency of a complementary heating system using solar energy and coal for a rural household building in northwest China was analyzed. The prototype model shows that the average thermal efficiency of the stove is 38%, which means that the portion of energy that enters into the building from solar energy and coal is 60.5% and 39.5%, respectively. A relational expression for the daily heat-collection capacity of the solar water heaters and the heat-loss coefficient of the storage tank was obtained. When compared to the reference solar water heater, it was found that the orientation of the solar water heaters and the shade between them have the largest influence on heat-collecting capacity. Regarding the solar energy projected onto the collecting area of the solar water heater, 49% was lost to optics and 23% to heat dissipation, with only 28% being used effectively. The mismatch between the working temperature of the radiator and the collecting temperature of the solar water heater and the location of storage tanks are considered to be the main factors that led to the high heat loss.

Several items must be considered for a solar heating system applied to rural household buildings in the future. It can be seen from the above analysis that the portion of SI is 16% higher than that of SII, which is caused by the orientation of the solar water heaters and the shade between them. Despite the influence from each of not being distinguished clearly in this research, it is highly recommended to consider a reasonable design of the building originally, especially regarding the layout of solar collectors, as well as for employing the passive technique. On the other hand, the radiant floor heating technology seems to be common feature of new era. Such advanced techniques can decrease the collecting temperature of the solar collectors and improve the collecting efficiency. The work of this paper is of great significance for the next study of the influence of multifactor coupling on the heat-collecting performance of solar collectors. Continuous and stable use of solar energy to meet the user's multilevel energy demand, not only for the improvement of people's livelihood in northwest China and the protection of the ecological environment of great value but also of international renewable energy, has important academic value and significance.

## Figures and Tables

**Table 1:** The detailed parameters of instruments.

Measured parameters	Measuring instruments	Range	Precision
Temperature	Pt-100 temperature sensor	−50~100°C	±0.1°C
Flow rate for building	LWGY-20 turbine flowmeter	0.7~7.0 m^3^/h	±0.45%
Flow rate for digester	LWGY-15 turbine flowmeter	0.4~4.0 m^3^/h	±0.45%
Solar radiation	TBQ-2 pyranometer	0~2000 W/m^2^	±2%
Coals weight	Platform balance	-	±0.2 kg

**Table 2:** Durations and results for *q*_*hm*_ and *Q*_*hm*_ for per kg mass of coal.

*q*_*hm*_/W/m^2^			*Q*_*hm*_ for per kg mass of coal/MJ/kg		
Duration	*q*_*hm*_	*q*_*hm*,ave_	Duration	*Q*_*hm*_/(kg coal)	*Q*_*hm*,ave_/(kg coal)
17:00 pm on 12 to 2:30 am on 13 Dec, 2014	32.3		9 to 11 Dec, 2014	5.2	6.4
17:30 to 24:00 pm on 2nd Jan, 2015	42.1		4 Jan, 2015	5.9	
17:00 to 23:00 pm on 5 Jan, 2015	36.1	38.1	6 Jan, 2015	7.2	
17:00 to 23:00 pm on 5 Jan, 2015	44.0		17 Jan, 2015	7.5	
19:50 pm on 15 to 1:50 am on 16 Feb, 2015	36.1		27 to 28 Jan, 2015	7.1	
			31 Jan to 3 Feb, 2015	6.5	
			6 Feb, 2015	5.5	

**Table 3:** The heat loss coefficient of the storage tank.

Duration		*U*_*s*_/W/K	*U*_*s*,av_/W/K
2014-12-21	0:00–8:00	4.17	4.10
2014-12-26	0:00–8:00	4.70	
2015-01-30	0:00–8:00	4.38	
2015-02-11	0:00–8:00	3.77	
2015-02-14	0:00–8:00	3.59	
2015-02-15	0:00–8:00	4.01	
